# Supplementary material for: Enzymes Involved in Antioxidant and Detoxification Processes Present Changes in the Expression Levels of Their Coding Genes under the Stress Caused by the Presence of Antimony in Tomato
Source: Plants (Basel). 2024 Feb 23;13(5):609. doi: 10.3390/plants13050609 (PMC10934734; doi:10.3390/plants13050609)
Supplement: Supplementary file 1 [file plants-13-00609-s001.zip › plants-2764251-supplementary.pdf]

Supplementary Materials:

Supplementary Table S1. The Sb content of roots and leaves and the corresponding bioaccumulation factor (BF) and translocation factor (TF) values in tomato plants. A total of 15 plants were analysed for each treatment with three biological replicates.

Sample	Roots	Shoots	TF
Control	4.7±0.8	nd	nd
0.5 mM Sb	10832.4±460.5	336.7±26.7	0.033
1.0 mM Sb	13066.5±390.4	564.7±51.2	0.041

Supplementary Table S2. Primers used for synthesis of tomato cDNAs.

Gene	ID Gene	Primers (5´-3´)	Size (pb)
Actin	Solyc04g011500.2.1	GAAATAGCATAAGATGGCAGACG	159
		ATACCCACCATCACACCAGTAT	
β-tubulin (TUB)	Solyc04g081490.2.1	AACCTCCATTCAGGAGATGTTT	180
		TCTGCTGTAGCATCCTGGTATT	
APX	Solyc06g005150.3	TCTGGTTTTGAGGGACCTTG	113
		GCTTTGTCTGATGGCAACTG	
DHAR	Solyc05g054760.2	TGAGCTTGGCTCCAAAACTG	144
		CTTCAGCCTTGGTTTTCTGG	
GR_cyt	Solyc09g091840.3	AAAGTGTGGAGCAACCAAGG	86
		CTGAACGCATGGTCACAAAC	
GR_chl	Solyc09g065900.3	TAGCAAAGTTCTGGGCTTGC	84
		AACCCTGCTTTGACTGCAAC	
GSNOR	Solyc09g064370.3	TCCATTTCAGCTGGTGACTG	81
		AGGGAAGGAACTTGTGATCG	
SOD	Solyc02g021140.3	GGATTTGGCTTGTCTTGAGC	99
		CGATCAGGGGGATATCATTC	
GST	Solyc01g099590.3	TGGGCTCGTTTTGTTGATG	80
		CCCTCTGCTTTTGTTTCTCC	
GST TCHQD	Solyc04g057890.3	TTCCTTGAACAGCCAGAAGG	138
		ACAGGAACTTTCGCACTTGG	

Supplementary Table S3. Genes used for the bioinformatics study.

Gene	ID Gene	Location	Information
APX	Solyc06g005150.3	Chromosome 6: 170,183-173,377	Ascorbate peroxidase
DHAR	Solyc05g054760.2	Chromosome 5: 65,455,922-65,460,219	dehydroascorbate reductase 1 dhar1
GRcyt	Solyc09g091840.3	Chromosome 9: 71,517,454-71,527,429	Glutathione reductase, cytosolic
GRchl	Solyc09g065900.3	Chromosome 9: 64,425,969-64,432,963	Glutathione reductase
GSNOR	Solyc09g064370.3	Chromosome 9: 61,781,099-61,785,732	GSNOR (S-(hydroxymethyl)glutathione dehydrogenase)
SOD	Solyc02g021140.3	Chromosome 2: 22,261,065-22,263,375	Superoxide dismutase
GST	Solyc01g099590.3	Chromosome 1: 89,654,694-89,655,730	Glutathione-S-transferase
GST TCHQD	Solyc04g057890.3	Chromosome 4: 54,949,331-54,955,349 reverse strand.	GST 2 Glutathione S-transferase TCHQD
